# A sound approach to stay on the ball—a review of scrotal pathologies on ultrasound imaging

**DOI:** 10.1093/bjr/tqag063

**Published:** 2026-03-17

**Authors:** Jeffrey Lam Shin Cheung, Mousumi Bhaduri

**Affiliations:** Medical Imaging Department, Victoria Hospital, London, ON N6A 5W9, Canada

**Keywords:** scrotum, testicle, ultrasound, testicular torsion, epididymitis, seminoma

## Abstract

Scrotal ultrasound imaging findings range from benign anatomical variants to surgical emergencies. Scrotal assessments can be daunting for clinicians who are unfamiliar with the typical anatomy and expected imaging characteristics of common pathologies. This pictorial review highlights key anatomical considerations and essential tips for scrotal ultrasound imaging. A practical approach to scrotal pathologies is discussed and supplemented with a plethora of ultrasound imaging examples. Particular emphasis is placed on describing the clinical presentation, imaging findings, and recommended management for: infectious/inflammatory conditions, vascular anomalies, sequelae of trauma, cystic lesions, and common testicular neoplasms.

## Introduction

Scrotal conditions range from benign anatomical variants to sequela of trauma to neoplasms among other possibilities. A framework to differentiate these is key for appropriately recommending conservative versus surgical management.

Medical imaging is essential in diagnosing scrotal conditions. Ultrasound remains the first line imaging modality due to its accessibility, efficiency, cost-effectiveness, and high imaging quality.^1–3^ Unfortunately, interpreting ultrasound images can be challenging for inexperienced clinicians who lack familiarity with the varying presentation states for scrotal pathologies.

**Figure 1:**
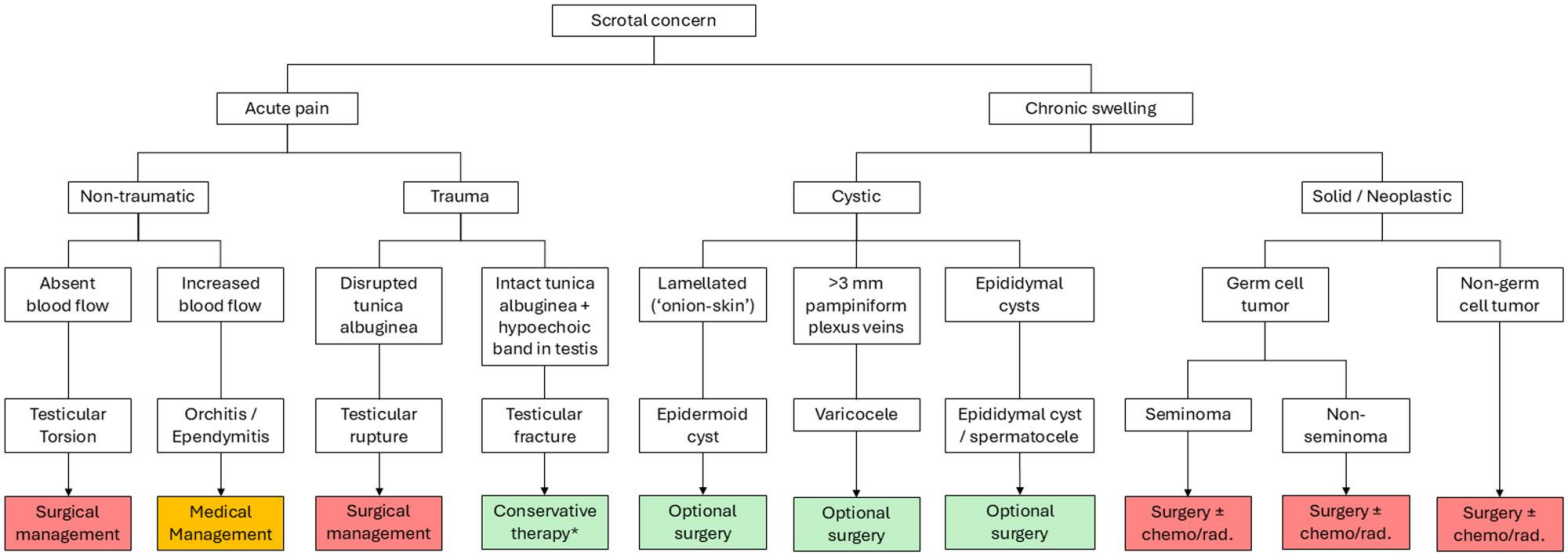
Simplified algorithm for common scrotal pathologies. *Testicular fracture treatment is typically conservative if vascularity is intact. Abbreviation: Chemo/rad. = chemotherapy/radiation therapy.

This review describes normal scrotal anatomy and key considerations for performing ultrasound scrotal imaging. Using ultrasound imaging examples from clinical cases, a framework for common scrotal pathologies is presented. Specifically, anatomical variants, infectious/inflammatory conditions, vascular pathology, traumatic findings, cystic lesions, and common neoplasms are discussed (Table 1 and Figure 1).

**Table 1:** An approach to common scrotal pathologies.

Category	Select differential diagnoses
Anatomical variants	Appendix testis/epididymis and scrotal pearl
	Rete testis
	Tubular ectasia of the epididymis
	Inguinal hernia^a^
Infectious/inflammatory	Epididymitis
	Orchitis
Vascular pathology	Varicocele
	Torsion
Trauma	Testicular fracture
	Testicular rupture
Cystic lesions/“-celes”	Hydrocele
	Hematocele
	Pyocele
	Epididymal cyst
	Spermatocele
	Epidermoid cyst
Neoplasms	Seminoma
	Nonseminomatous germ cell tumor
	Lymphoma
	Testicular adrenal rest tumor

a *Albeit* not a true anatomical variant, this is a relatively common mimicker of abnormal scrotal pathology.

## Testicular ultrasound imaging overview

Scrotal ultrasound imaging follows universal practices. A high frequency (7.5-15 MHz) linear array transducer optimizes the view of the superficial scrotal structures.^2–5^ Patients are typically positioned supine with a rolled towel between the thighs and draping over the thighs and abdomen.^1^ Images includes orthogonal sagittal and transverse views with transverse views allowing side-by-side comparisons of both testes. Finally, cine loops should be considered, especially when the radiologist reader cannot be present for complex cases.^2^

The advantages of ultrasound imaging include accessibility, expediency, non-invasiveness, and image quality.^1–3^ The ability to perform Doppler interrogation allows real-time assessment of vascular flow with dynamic repositioning and the Valsalva maneuver as needed.^1^ This is particularly helpful for assessing vascular pathologies.

Challenges of ultrasound include sonographer and patient variables. Sonographer experience is rate limiting in every step. For example, as there are no absolute standards for setting Doppler scales,^6^ an inappropriately selected Doppler scale may provide misleading results.^1^ On the patient side, large scrotal swelling may obscure the testes, though this can be partially mitigated with a curved transducer at a lower frequency. Patient discomfort, tenderness, guarding, movement, or non-compliance with repositioning may also limit image acquisition.^1^

## Testicular anatomy overview

The scrotal sac contains, protects, and regulates temperature for the testes.^4^ From superficial to deep, the scrotal layers include skin, the dartos muscle, the cremasteric muscle and fascia, and the internal spermatic fascia which is continuous with the abdominal transversalis fascia.^4^ The internal spermatic fascia contains the tunica vaginalis which consists of a visceral and parietal layer normally separated by 2-3 mL of fluid.^5^

Each testis is surrounded by a fibrous capsule called the tunica albuginea, which appears as a thin hyperechoic line on ultrasound.^2^^,^^6^ The 2 testes are separated by a median raphe and suspended by a spermatic cord. One testis (typically the left) may normally descend slightly lower than the other.^5^

The testes are paired ovoid structures responsible for spermatogenesis. On ultrasound, the testes normally appear homogeneously echogenic. A normal adult testis measures about 5 × 3 × 2 cm and regresses in size with age.^1^^,^^2^^,^^4–7^ Each testis contains numerous lobules composed of seminiferous tubules, the site of spermatogenesis.^5^ The seminiferous tubules transition into tubuli recti which become the rete testes before leading to the epididymis.^1^

The epididymis, which derives from the Wolfian ducts, runs along the outer testis and stores spermatozoa.^4^ It attaches to the testis at the hilum and is supported by the mediastinum testis, a hyperechoic band on ultrasound. The epididymis is hypoechoic relative to the testis and measures 6-7 cm long. From superior to posterolateral, the epididymis segments include the head (the largest portion measuring 10-12 mm in diameter), body, and tail.^6^ From the tail, sperm travels to the vas deferens.

Scrotal venous draining is provided by the pampiniform venous plexus, a network of veins. On Doppler interrogation, these tubular structures demonstrate vascular flow.^4^ The right testicular vein drains directly to the right inferior vena cava, while the left testicular vein orients vertically and drains into the left renal vein. This asymmetry predisposes the left side to vascular pathologies such as varicoceles.^8^

## Anatomical variants

### Appendix testis and epididymis

**Figure 2:**
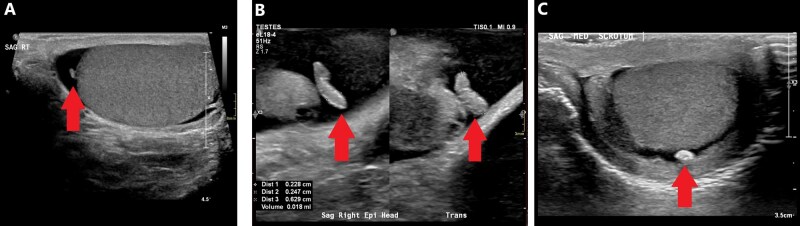
Appendix testis and epididymis and scrotal pearl. (A) The red arrow points to an isoechoic ovoid structure protruding from the testis, consistent with an appendix testis. (B) The red arrow points to an isoechoic tubular structure protruding from the epididymis head, consistent with an appendix epididymis. (C) The red arrow points to a hyperechoic extra-testicular scrotal structure, consistent with a scrotal pearl.

A remnant of the Mullerian duct may result in an appendix testis (also called an “appendage”) protruding from the testis.^6^ Similarly, a remnant of the Wolfian duct may result in an appendix epididymis, typically protruding from the epididymis head. Appendices are typically small isoechoic ovoid or tubular structure (Figure 2).^9^ Torsion of an appendix testis/epididymis can potentially cause pain. Dead appendix tissue can detach from the testis/epididymis and calcify resulting in a round hyperechoic scrotal pearl (also called a “scrotolith”), which is of no clinical concern (Figure 2).^5^

### Rete testis

The rete testis bridges the testis and epididymis and is supported by the mediastinum testis. On ultrasound, the rete testis can range from an ill-defined hypoechoic region in the testicular hilum to a coarse tubular appearance with finger-like projections entering the testicular parenchyma (Figure S1).^10^^,^^11^ Dilatation of the rete testis ducts is uncommon and termed tubular ectasia of the rete testis.^12^

### Tubular ectasia of the epididymis

**Figure 3:**
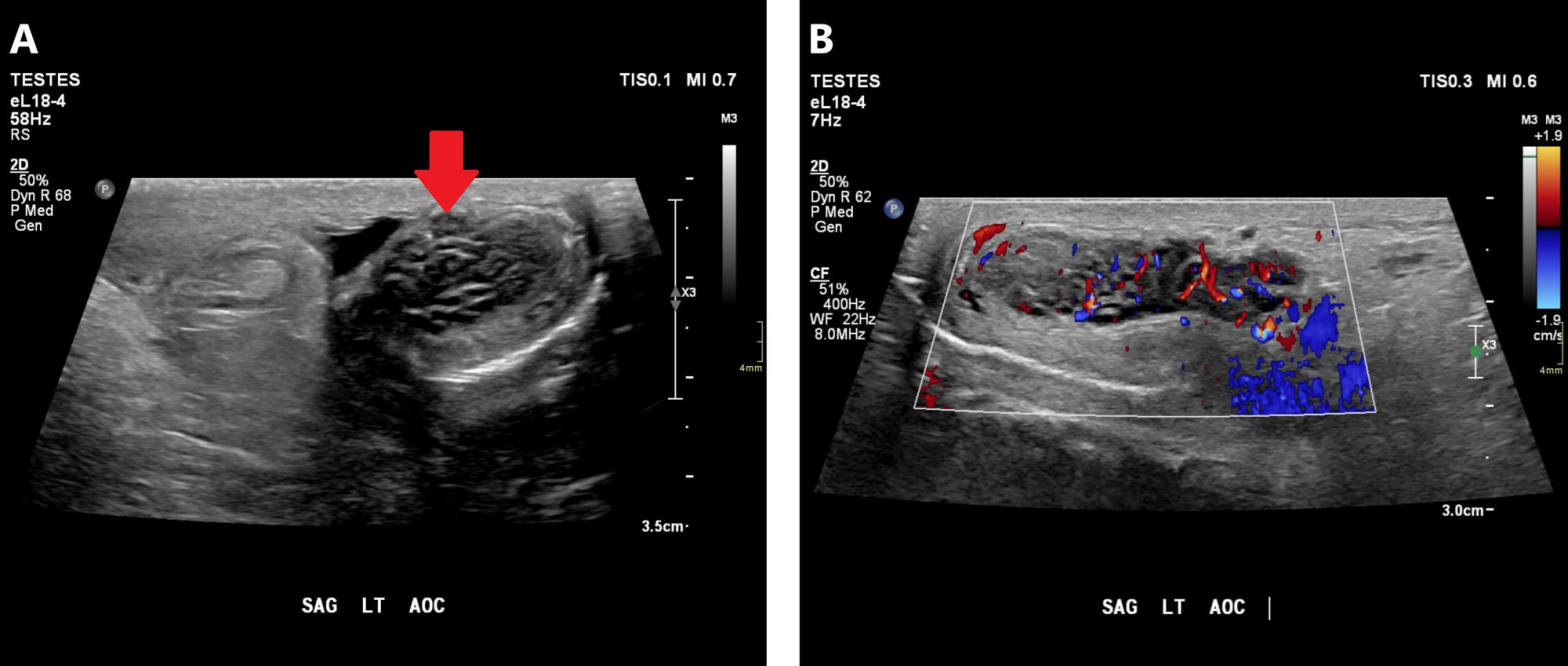
Tubal ectasia of the epididymis. (A) The epididymis demonstrates cystic, tubular dilatation (red arrow). (B) Doppler interrogation demonstrates normal vascularity pointing away from an inflammatory process such as epididymitis.

Tubular ectasia of the epididymis classically appears as cystic, tubular dilatation of the epididymis (Figure 3).^9^ This is frequently bilateral and asymmetric. Unlike epididymitis, tubular ectasia demonstrates normal blood flow or hypovascularity on Doppler interrogation.^13^ Predisposing factors include older age and prior vasectomy which is thought to cause epididymal outflow tract obstruction.^9^^,^^13^

### Inguinal hernia

Although not a normal anatomical variant, inguinal hernias are common with a male lifetime incidence of 30%.^14^ A direct inguinal hernia occurs medial to the inferior epigastric vasculature, whereas an indirect inguinal hernia occurs lateral to the inferior epigastric vasculature through the inguinal ring. Indirect inguinal hernias can potentially contain abdominal contents.^14^ Ultrasound images may show omental fat, small bowel, colon, or even bladder herniating into the scrotal sac (Figure S2). Cine clips may help demonstrate peristaltic movement of herniated bowel.^14^

## Infectious and inflammatory pathology

### Epididymitis

Epididymitis (epididymis inflammation) is a common cause of scrotal pain with over 500 000 new cases in North America annually.^9^ The classic clinical presentation involves scrotal swelling and gradual onset of scrotal pain.^12^ Symptoms may be unilateral or spread bilaterally.^12^ Lower urinary tract infectious symptoms including fever, urinary frequency, dysuria, and hematuria may also be present.^12^ The typical demographic is 18- to 35-year-old sexually active adults as sexually transmitted bacteria including *Neisseria gonorrhea* and *Chlamydia trachomatis* are the most common infectious causes of epididymitis.^12^ Other bacterial etiologies include *Escherichia coli* and *Proteus mirabilis* from prior urinary tract infection.^9^^,^^12^ Less common non-infectious causes include vasculitides and medication reaction such as from amiodarone.^12^^,^^15^

**Figure 4:**
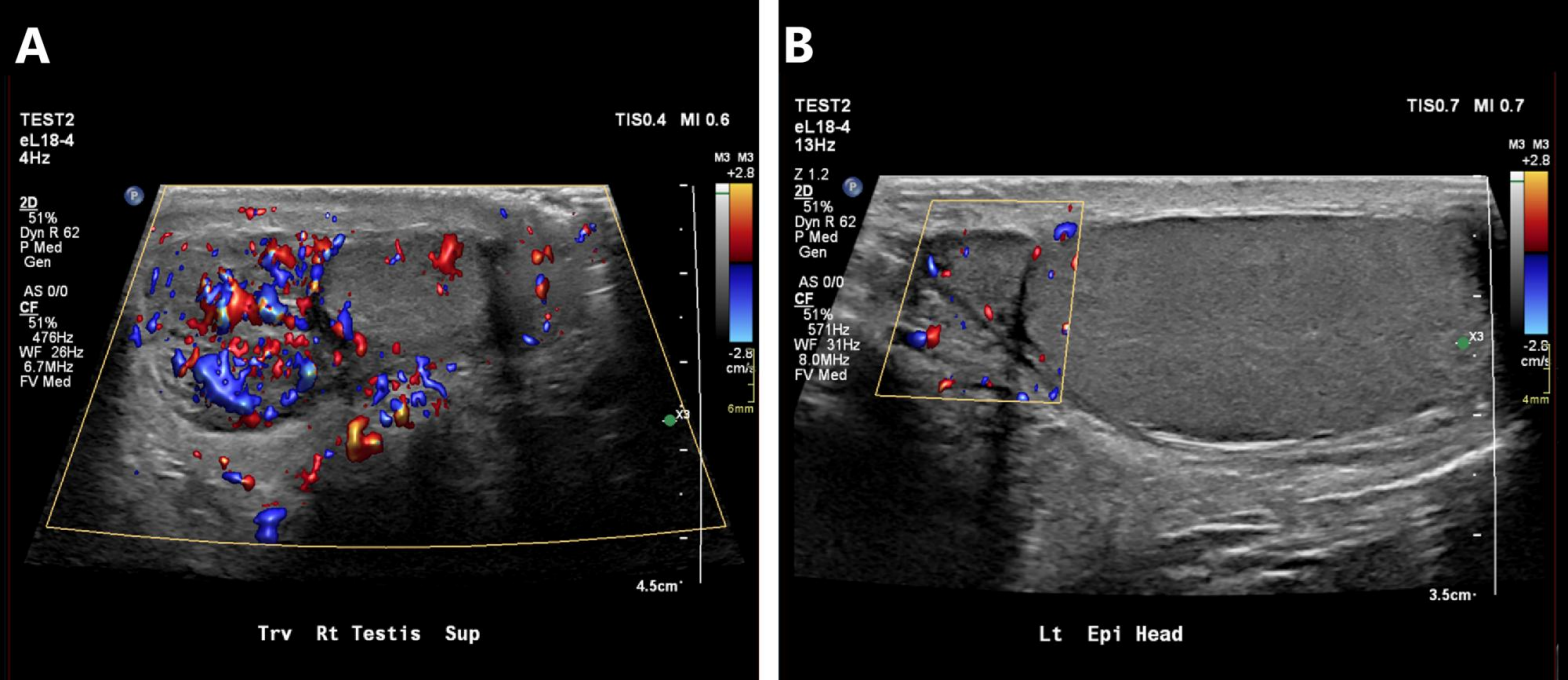
Right-sided epididymitis. (A) The right epididymis is enlarged and heterogeneous testis with marked hyperemia on Doppler interrogation. (B) For comparison, the contralateral epididymis is more normal in size and blood flow.

On ultrasound, epididymitis classically presents as a thickened epididymis with hyperemia on Doppler interrogation (Figure 4).^9^^,^^12^ Simultaneous transverse imaging comparing both epidymi can reliably confirm hyperemia. Secondary signs include a reactive hydrocele which presents as an anechoic fluid collection, sometimes with echogenic debris reflecting infectious fluid content.^12^ Ultrasound is 70% sensitive and 88% specific for identifying epididymitis.^12^

Epididymitis is typically treated on an outpatient basis with the primary goal of treating the underlying cause. For gonorrhea and chlamydia infection, antibiotics such as ceftriaxone and doxycycline are used.^12^ For enteric organisms, ofloxacin or levofloxacin may be preferred.^12^ Local antibiotic protocols may vary.

### Orchitis

Orchitis (testicular inflammation) is commonly secondary to infectious epididymitis which has spread to the testes (epididymo-orchitis).^12^ Isolated orchitis without epididymitis is rare and may occur in mumps. Up to 20% of mumps cases have associated orchitis.^12^ The typical clinical presentation includes scrotal swelling and testicular pain which tends to be more acute relative to epididymitis.^12^

**Figure 5:**
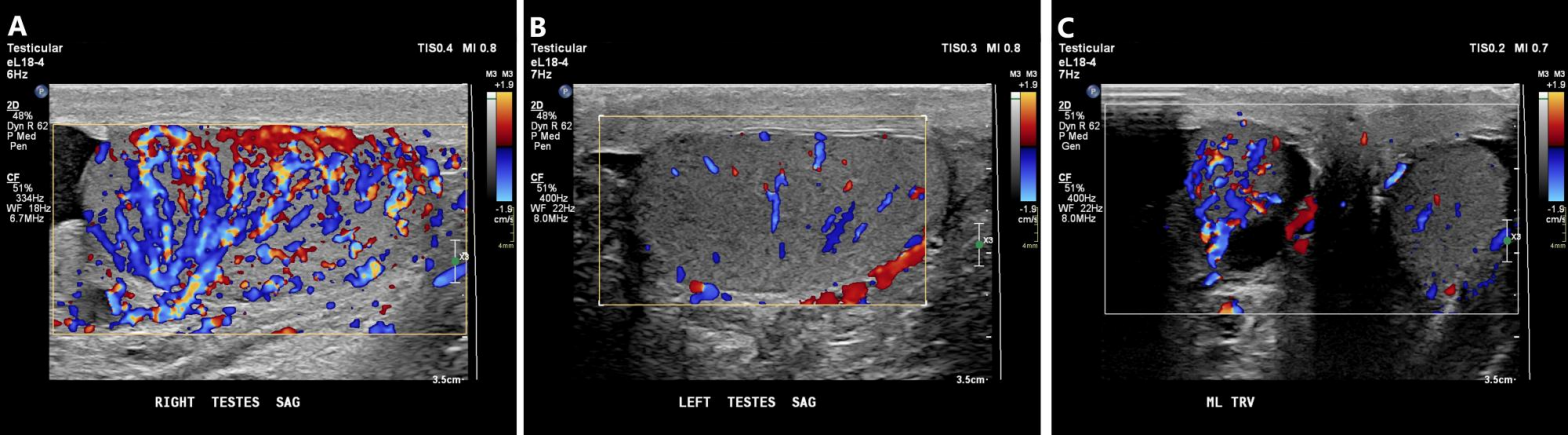
Orchitis of the right testis. (A) The right testis is enlarged and demonstrates hyperemia on Doppler interrogation. (B) The contralateral left testis has a normal appearance and blood flow. (C) When both testes are viewed simultaneously, the right testicular hyperemia is more easily appreciated.

On ultrasound, orchitis typically shows testicular enlargement due to edema.^15^ The increased fluid content may cause the affected testis to appear slightly hypoechoic compared to normal.^15^ As with epididymitis, Doppler interrogation is key to demonstrate hyperemia denoting an inflammatory process.^15^ Ideally, both testes should be imaged simultaneously to have the non-affected testis act as a control (Figure 5). A reactive hydrocele may also present and have internal debris and echoes indicating infectious content.^15^

While epididymo-orchitis is managed similarly to epididymitis alone, isolated orchitis is treated more conservatively with a focus on symptomatic relief. Mumps orchitis is self-resolving, usually within 10 days.^12^

## Vascular pathology

### Varicocele

Varicoceles are abnormal dilatation of the pampiniform plexus and occur in approximately 15% of males.^8^ Varicoceles are frequently identified during infertility workup, occurring in up to 40% of infertile men.^8^^,^^16^ When symptomatic, varicoceles may have a “bag of worms” sensation potentially causing dull aching or throbbing scrotal pain.^16^ The vertical course of the left testicular vein predisposes left-sided varicoceles.^8^

**Figure 6:**
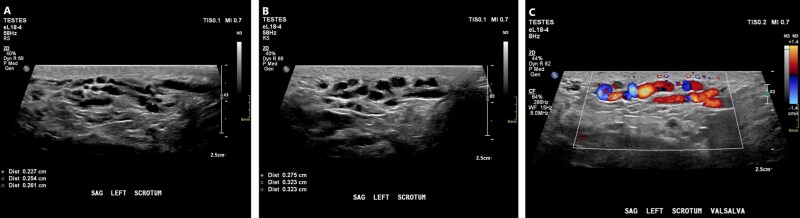
Left sided varicocele. (A) Within the left scrotum, there are enlarged tubular structures measuring up to 2.6 mm in diameter. (B) With Valsalva maneuver, the tubular structures dilate up to 3.2 mm. (C) There is flow on Doppler interrogation confirming that these tubular structures represent veins of the pampiniform plexus.

Varicocele ultrasound features match physical exam findings. The pampiniform plexus appears as abnormally dilated tubular structures with further dilatation during Valsalva maneuver.^8^ Some authors have proposed a vein diameter cut-off of 2.5 mm at rest and 3.0 mm with Valsalva pressure.^8^ Doppler interrogation is necessary to demonstrate blood flow and confirm that the tubular structures represent pampiniform plexus veins (Figure 6). Ultrasound is 97% sensitive and 94% specific.^8^

Varicocele treatment is generally recommended for infertility.^16^ A varicolectomy can be performed by different approaches including retroperitoneally, microscopically with a subinguinal route, or laparoscopically. For poor surgical candidates, percutaneous angiography may be a preferred alternative.^16^

### Torsion

Testicular torsion is a surgical emergency and important consideration for acute onset scrotal pain. This occurs when the spermatic cord twists on itself thus cutting off testicular arterial blood supply.^17^ The bell clapper deformity, a high riding spermatic cord (Figure S3), enables cord mobility and is a risk factor.^17^ Neonates and puberty-aged males are at highest risk.^18^ Unlike epididymitis, torsion is associated with an absent cremasteric reflex (testicular elevation elicited by stroking the medial thigh skin) and negative Prehn’s sign (pain relief with testis elevation).^12^^,^^17^

**Figure 7:**
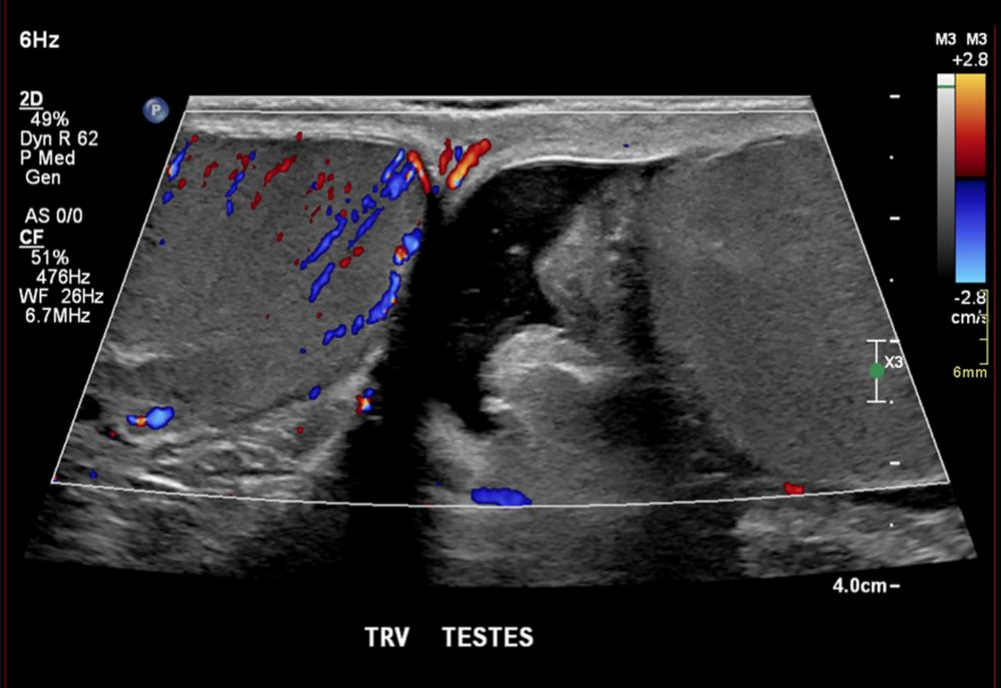
Left-sided testicular torsion. Doppler interrogation demonstrates a complete lack of blood flow in the mildly enlarged, edematous left testis (right side of the image) relative to the normal contralateral side.

Although testicular torsion is a clinical diagnosis, ultrasound helps in equivocal cases. Early ultrasound findings may demonstrate normal testicular anatomy. Later findings include a heterogeneous and hypoechoic appearing testis corresponding with the side of pain.^17^ Doppler assessment of blood flow and concurrent comparison to the contralateral unaffected testis is key.^17^^,^^18^ Early findings may paradoxically show hyperemia due to venous dilatation or rebound perfusion following de-torsion.^17^ Advanced findings are classic for absent blood flow (Figure 7), potentially with increased para-testicular collateral flow.^17^^,^^18^ Ultrasound generally has a sensitivity of 63%-100% and specificity of 80%-100%.^17^^,^^19^ Demonstration of the whirlpool sign, direct visualization of the twisted spermatic cord, has a sensitivity of 92% and specificity of 99%.^18^^,^^19^ As with many testicular pathologies, a reactive hydrocele may be present.

Recognition of testicular torsion requires urgent urology referral for time sensitive surgical management.^12^ Optimal outcomes occur with earlier intervention. Testicular salvage rates are 100% within 6 h, 75% within 12 h, and 50% within 12-24 h of symptom onset.^18^ Delayed treatment risks causing irreparable testicular damage, infertility, and cosmetic deformity.^19^

## Trauma

### Testicular fracture and rupture

Testicular injuries account for less than 1% of all trauma-related injuries.^7^^,^^20^ Injuries are usually unilateral, with only 1.5% of testicular injuries being bilateral.^7^ Two potential outcomes include testicular fracture (disruption in the testicular parenchyma) and testicular rupture (disruption of the tunica albuginea).^20^

**Figure 8:**
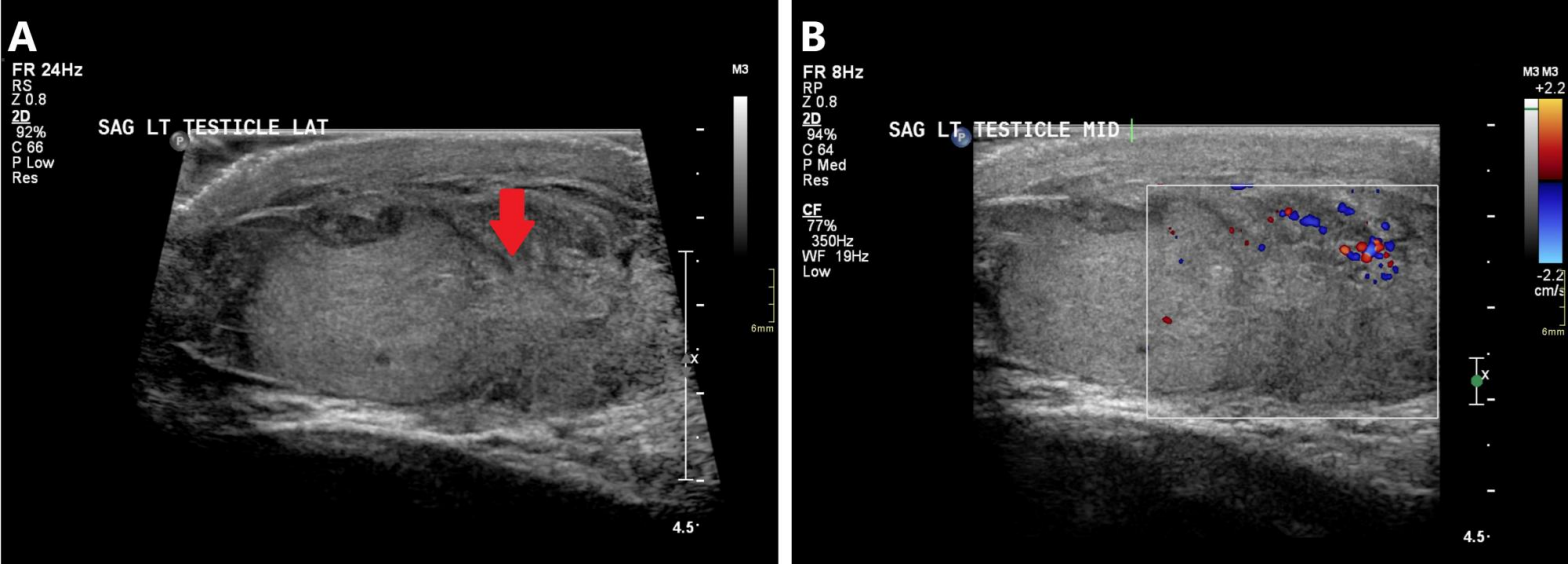
Left testicular rupture. (A) There is a disruption of the tunica albuginea (red arrow) with a loss of the normal ovoid shape of the left testis. (B) Doppler interrogation shows relative avascularity in the surrounding heterogeneous collection consistent with a hematocele.

Ultrasound is essential for characterizing testicular injuries. A testicular fracture appears as a linear hypoechoic band within the testis parenchyma.^20^^,^^21^ A testicular rupture demonstrates discontinuity of the echogenic tunica albuginea, frequently with extruded testicular contents (Figure 8).^20^^,^^21^ Resultantly, there may be a para-testicular anechoic hydrocele or hematocele. An acute hematocele appears echogenic and becomes progressively hypoechoic and heterogeneous over time.^20^^,^^21^ Pressure from large fluid collections may suppress blood flow and imaging may mimic a testicular torsion on Doppler interrogation.^20^ Doppler assessment is not strictly necessary for diagnosing testicular injury, but it assists with assessing injury severity, vascular integrity, and prognosis.^7^^,^^20^^,^^21^ Ultrasound is 64%-100% sensitive and 65%-93.5% specific for identifying testicular trauma.^7^

Treatment differs for testicular fractures and ruptures. Testicular fracture alone can be managed conservatively if vascularity is preserved.^20^ Testicular rupture, however, requires urgent urology consultation for surgical repair.^20^ Outcomes are generally favorable with a 90% salvageable rate when treatment is performed within 72 h from injury onset.^7^ In cases of severe injury where a testis is no longer salvageable, orchiectomy may be considered.^7^

## “-Celes” and cystic lesions

### Hydrocele, hematocele, and pyocele

A hydrocele is excess fluid within the tunica vaginalis, surpassing the normal 3 mL physiologic limit.^22^ Most cases are idiopathic and clinically present as painless scrotal swelling.^23^ Additional symptoms may reflect an underlying inflammatory, infectious, traumatic, or neoplastic cause.^22^^,^^24^ A hydrocele containing blood is termed a hematocele.^22^ Similarly, a hydrocele containing infectious content is termed a pyocele.^24^

**Figure 9:**
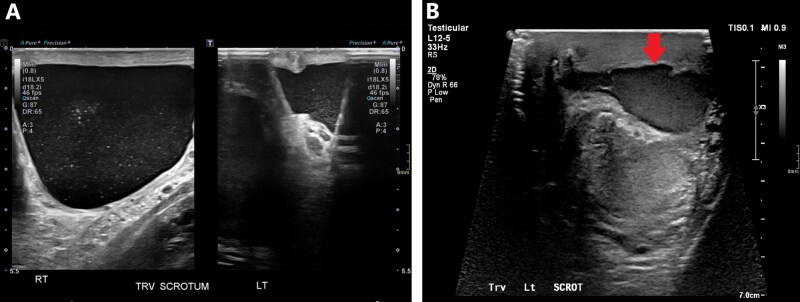
Hydrocele vs. hematocele. (A) Within the scrotum, there is a large volume of anechoic fluid. This hydrocele contains echogenic debris which can potentially reflect infectious or cellular material. (B) In another patient, there is disruption of the left testicular tunica albuginea and an adjacent fluid collection (red arrow) with layering hyperechoic material consistent with a post-traumatic hematocele.

Ultrasound appearances of hydroceles vary based on the fluid content. Simple hydroceles are anechoic para-testicular avascular collections.^22^^,^^24^ Occasionally, debris may be present within the fluid and reflect cellular content (Figure 9). Over time, chronic hydroceles may develop septations, and larger collections may apply pressure reducing testicular arterial blood flow and mimicking testicular torsion on Doppler interrogation.^22^ A pyocele may develop gas appearing as bright echoes.^24^ A hematocele appears as a heterogeneous hypo-to-anechoic collection, and fluid-fluid levels may develop as hemoglobin breaks down (Figure 9).^22^ Doppler interrogation is helpful in assessing para-testicular collections to verify avascularity.^22^ Ultrasound also assists in searching for an underlying etiology such as a testicular mass or inflammation.^23^

Management of hydroceles depends on the fluid volume and clinical symptoms. Small asymptomatic collections and hydroceles secondary to varicolectomy are managed with a watch and wait approach.^23^ Additionally, most hematoceles self-resolve with time. Fluid aspiration is reserved for larger collections that exert pressure and suppress testicular arterial blood flow.^22^ Aspiration is done by hydrocelectomy with a scrotal incision and generally has favorable outcomes.^23^

### Epididymal cyst and spermatocele

Epididymal cysts are common and incidental in up to 40% of asymptomatic patients.^22^^,^^24^ These are generally small, but when sufficiently large, they may alternatively represent dilated efferent tubules known as a spermatocele.^22^^,^^24^

**Figure 10:**
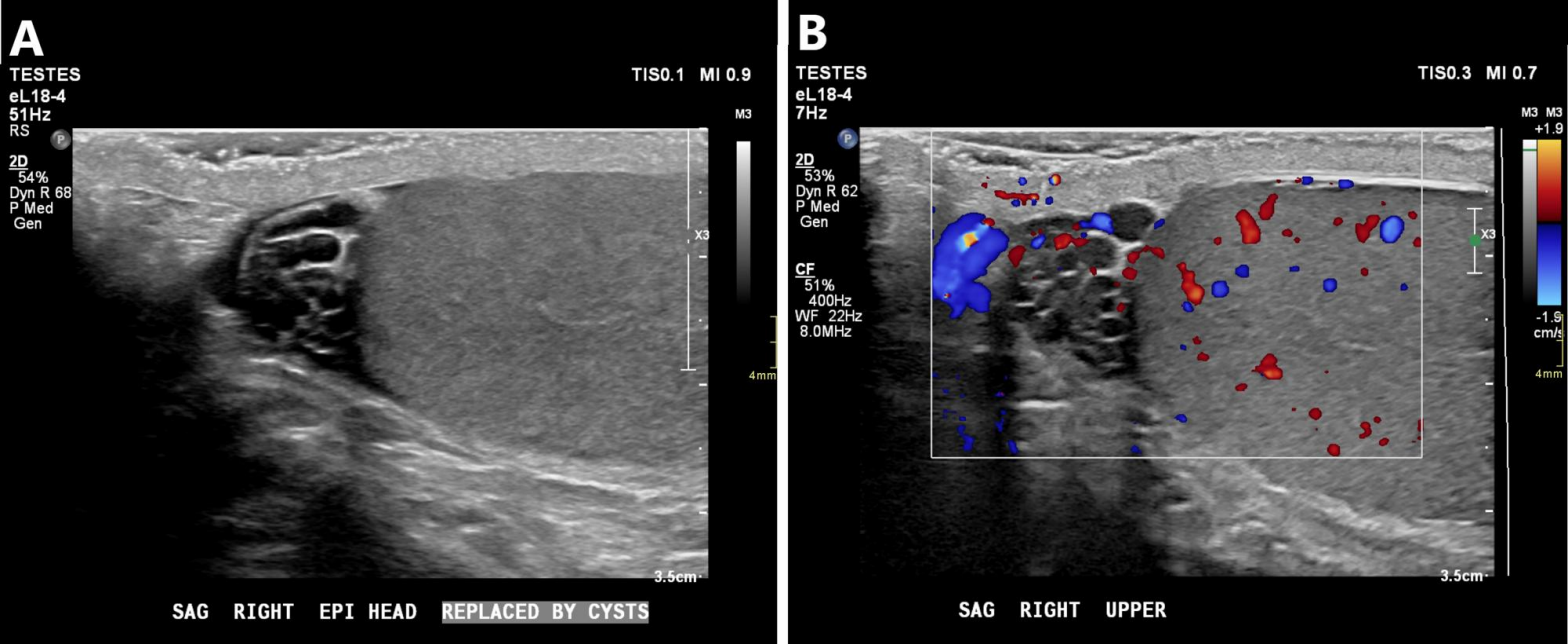
Spermatocele. (A) The epididymal head is nearly completely replaced by cystic lesions with echogenic debris. (B) Doppler interrogation proves that the cystic structures are not blood vessels.

Ultrasound can help differentiate epididymal cysts from spermatoceles. Epididymal cysts are most common at the head of the epididymis and appear as cysts elsewhere in the body: defined avascular anechoic fluid collections with a posterior echogenic wall and posterior acoustic enhancement.^22^^,^^24^ Unlike typical simple cysts, spermatoceles classically contain echogenic internal debris reflecting sperm, protein, and/or neutrophils (Figure 10).^22^^,^^25^ A spermatocele is also more likely to displace the testis due to its increased size.^25^^,^^26^ Doppler interrogation should demonstrate avascularity to rule out a vascular lesion.

Most epididymal cysts and spermatoceles are left alone with no further treatment required. Large symptomatic cysts and spermatoceles may undergo surgical resection through spermatocelectomy.^26^

### Epidermoid cyst

Epidermoid cysts (synonymous with keratocysts) are benign germ cell tumors.^23^^,^^27^ These are rare, comprising only 1.5% of all testicular neoplasms.^27^ Patients are typically young adults between the age of 16-34 and present with a painless scrotal mass, more frequently on the right side.^27^

**Figure 11:**
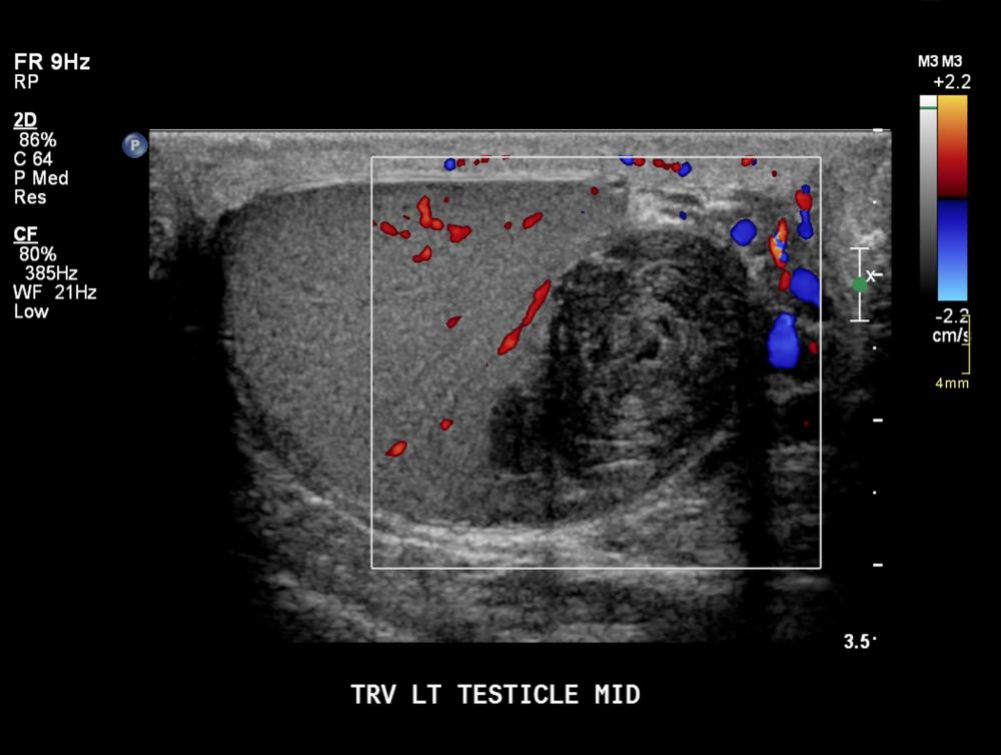
Epidermoid cyst. Within the left testis, there is an avascular mass with alternating hypoechoic and hyperechoic concentric rings (onion-ring appearance).

Epidermoid cysts vary in size, usually ranging from 1 to 3 cm, and have characteristic ultrasound findings.^23^ Up to 60% of cases have a lamellated “onion-ring” pattern demonstrating alternating hypoechoic and hyperechoic rings (Figure 11).^27^ Alternatively, epidermoid cysts may have a target appearance with a central hyperechoic region surrounded by a hypoechoic halo.^23^ On Doppler interrogation, these cysts are avascular centrally.^23^^,^^27^

Local excision is the preferred management for epidermoid cysts.^27^ To date, recurrence following excision has not been reported.^27^ Orchiectomy may be considered in cases of large epidermoid cysts with little remaining normal testicular parenchyma.^27^

## Neoplasm

Despite accounting for only 1% of all male cancers, testicular tumors are the most common cancer in the 15- to 34-year-old age range.^28^ Germ cell tumors (GCTs) comprise 95% of all testicular tumors, while the remaining 5% consist mostly of sex cord stromal tumors such as Leydig cell, Sertoli cell, granulosa cell, and thecoma-fibroma tumors.^29^ Other types of testicular tumors include lymphoma, leukemia, sarcoma, fibroma, and metastases most commonly from melanoma and prostate, lung, kidney, and colon cancer.^29^ Cryptorchidism (undescended testes) is a well-known risk factor for testicular tumors.^29^

As testicular neoplasms typically present as solid lesions with vascularity on ultrasound, there may be overlapping findings with other inflammatory processes.^28^ Contrast-enhanced ultrasound (CEUS) may help troubleshoot cases where vascularity is questioned to reflect an inflammatory process such as orchitis. CEUS is a relatively newly developed tool whereby microbubbles are intravenously injected and detected with ultrasound as a means of assessing perfusion.^30^ Generally, tumors demonstrate rapid wash-in and wash-out kinetics unlike non-neoplastic inflammatory/infectious processes.^30^

Although standard ultrasound cannot differentiate specific tumor histologies, elastography can be an adjunct tool for differentiating benign from malignant lesions. Elastography ultrasound can measure tissue stiffness, and early data suggests that malignant tumors tend to be stiffer compared to benign lesions.^31^

### Seminoma

As 50% of all GCTs are seminomas, seminomas are the most common testicular tumor.^28^^,^^29^^,^^32^ The average age at presentation is 40, which is slightly older than other GCTs.^32^ Patients typically present with painless scrotal swelling or a palpable lump.^28^ Up to 25% of cases report scrotal pain.

**Figure 12:**
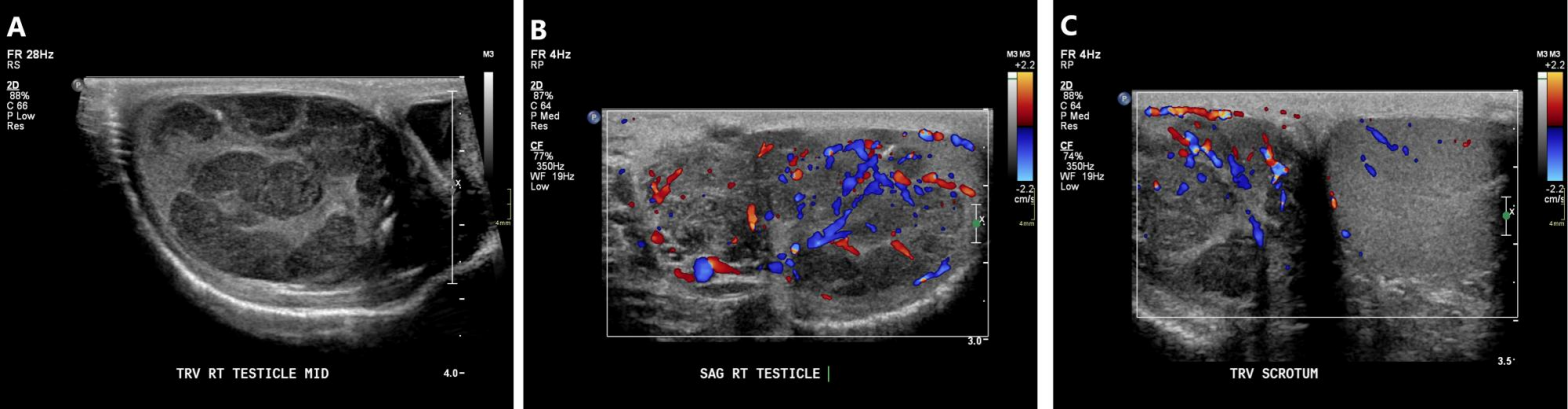
Right testicular seminoma. (A) There are multiple defined hypoechoic lesions within the right testis. (B) Doppler interrogation demonstrates vascularity indicating that these are not cystic lesions. (C) Concurrent comparison with the normal left testis accentuates the aforementioned findings.

As with other testicular tumors, ultrasound alone cannot definitively diagnose seminomas, but findings may be suggestive.^29^ Seminomas vary in size from a few millimeters to large masses that essentially replace the testis.^32^ Seminomas are usually homogeneously hypoechoic and may have microlithiasis or macrocalcification (Figure 12).^29^^,^^32^^,^^33^ Heterogeneity is occasionally observed when necrotic changes have occurred. On Doppler interrogation, seminomas typically demonstrate vascularity. A seminoma may appear avascular if it has burned-out, signifying that the tumor has outgrown its blood supply.^33^

Orchiectomy is the gold standard for treating seminomas.^34^ Testicular sparing surgery may be considered for smaller masses, which are more likely to be benign or early stage.^34^ Compared to other GCTs, seminomas tend to have a better prognosis with radiation and chemotherapy.^32^ The efficacy of chemotherapy for testicular tumors has been debated due to the theoretical ability of the blood-testis barrier to inhibit chemotherapy transportation.^35^

### Nonseminomatous germ cell tumor

Nonseminomatous GCT is an umbrella term encompassing a broad variety of tumors such as embryonal carcinoma, yolk sac tumor, choriocarcinoma, teratoma, and mixed germ cell tumor. Patients typically present with painless scrotal swelling or a palpable lump.^28^ Unlike seminomas, the average age of presentation for nonseminomatous GCT is younger at 30 years.^32^ Also different from seminomas, many nonseminomatous GCTs have elevated alpha fetoprotein serum levels (excluding choriocarcinoma which is classically associated with elevated beta human chorionic gonadotropin).^36^

**Figure 13:**
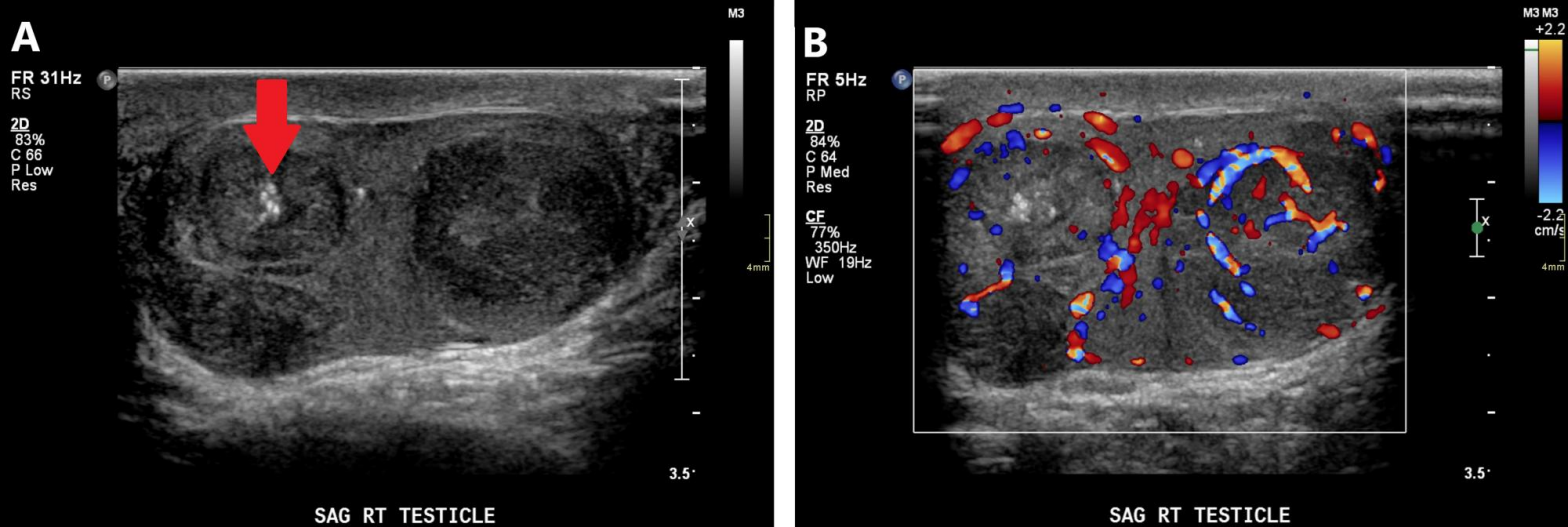
Nonseminomatous germ cell tumor. (A) Within the right testis, there are at least 3 heterogeneous predominantly hypoechoic lesions with irregular borders. One of the lesions contains central hyperechoic foci (red arrow) potentially representing hemorrhage, calcification, or fibrosis. (B) Doppler interrogation demonstrates vascularity indicating that these are not cystic lesions.

**Figure 14:**
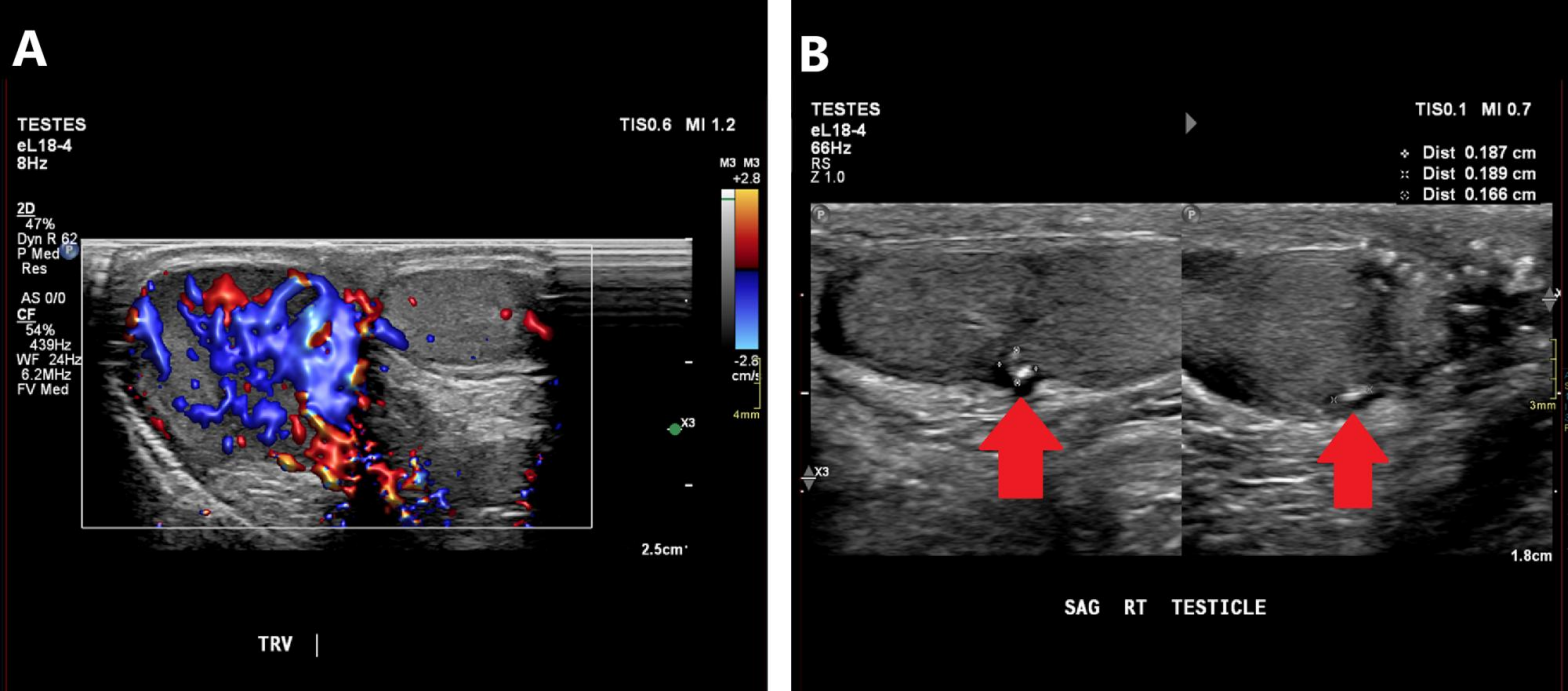
Burned-out tumor. (A) There is heterogeneity and markedly increased vascularity of the right testis in a patient with known leukemia. Scrotal findings are favored to represent leukemia infiltrate. (B) A follow-up scan 3 months later demonstrates resolution of the previous hyperemia with a residual calcified mass (red arrow) consistent with a burned-out tumor.

Nonseminomatous GCTs have several overlapping ultrasound features. These frequently present as heterogeneous masses with irregular margins.^32^ They occasionally contain cystic components and echogenic foci reflecting hemorrhage, calcification, or fibrosis (Figure 13).^32^ Similar to a seminoma, a nonseminomatous GCT may become burned-out appearing as a small avascular mass or focal calcification (Figure 14).^29^

Orchiectomy is the gold standard for treating nonseminomatous GCTs.^34^ If chemotherapy is used, post-treatment ultrasound may show scar tissue that cannot be reliably distinguished from residual tumor. Resultantly, delayed orchiectomy is still recommended following chemotherapy.^34^^,^^35^

### Lymphoma

Although testicular lymphoma is rare, it is the most common testicular cancer in males over 60 years old.^32^ Secondary lymphoma is more common than primary, and non-Hodgkin lymphoma is more common than Hodgkin.^29^^,^^32^ Patients typically present with a firm painless palpable scrotal mass.^37^ Concurrent constitutional symptoms, such as weight loss and fatigue, suggest systemic disease involvement.^37^

**Figure 15:**
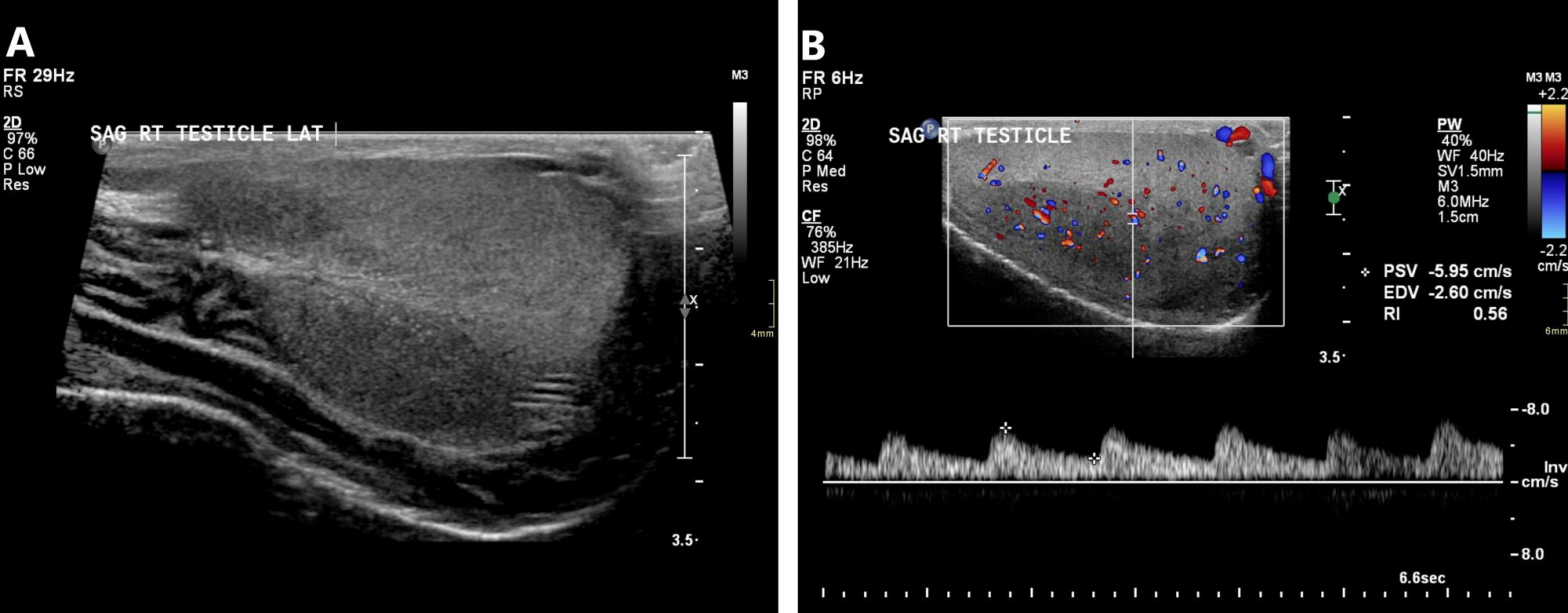
Right testicular lymphoma. (A) Within the right testis, there is a suspicious hypoechoic region posterior to the mediastinum testis. (B) Doppler interrogation reveals mild hyperemia in this hypoechoic region.

On ultrasound, lymphoma typically presents as 1 or multiple intratesticular lesions that are hypoechoic relative to testicular parenchyma. Bilateral involvement is present in up to 35% of cases.^28^^,^^32^^,^^37^ On Doppler interrogation, these masses tend to be vascular and hyperemic (Figure 15).^29^^,^^37^ As ultrasound findings are not specific, clinical history and patient demographics are helpful for recognizing this disease. Prior imaging demonstrating systemic disease involvement can help support a diagnosis.^37^

Testicular lymphoma treatment generally involves a combination of orchiectomy, radiation, and chemotherapy.^37^ Recurrence following chemotherapy is relatively common and thought to be due to the blood-testis barrier limiting medication from reaching the intratesticular lymphoma.^28^^,^^32^^,^^37^

### Testicular adrenal rest tumor

Although the exact etiology is currently unknown, testicular adrenal rest tumors (TARTs) are postulated to represent ectopic adrenal cells in the testes.^38^ Approximately 40% of patients with congenital adrenal hyperplasia have a TART.^38^^,^^39^ As TARTs respond to adrenocorticotropic hormone release, detection also increases with age.^38^ A TART may not be detectable clinically as they are frequently asymptomatic and rarely cause scrotal pain.^38^^,^^39^ TARTs tend to grow adjacent to the mediastinum testis and can resultantly cause obstruction of the seminiferous tubules leading to infertility.^40^

On ultrasound, TARTs have variable presentations and lack specific features. Most commonly, a TART appears as a hypoechoic circumscribed lesion with hyperechoic foci near the mediastinum testis.^38^^,^^39^ They are bilateral in 77% of cases and demonstrate vascularity on Doppler interrogation (Figure S4).^38^

There are currently no definite guidelines for managing TARTs. Exogenous glucocorticoid is often administrated to reduce adrenocorticotropic hormone production through negative feedback.^38^^,^^39^ Surgical intervention may help resolve pain associated with a TART but will not reverse infertility.^38^^,^^40^

## Conclusion

Ultrasound imaging of the scrotum is an indispensable clinical adjunct. Familiarity with normal and variant scrotal anatomy helps clinicians recognize and better understand the pathology of abnormal findings. When encountering an unfamiliar scrotal ultrasound finding, clinicians can use a basic framework to assess whether the lesion represents an infectious/inflammatory condition, vascular anomaly, sequela of trauma, cystic lesion, or neoplasm.

## Pitfalls and practical teaching points

1. Check for optimized Doppler settings. If the pulse repetition frequency is too high, this may result in a false positive. Alternatively, if the pulse repetition frequency is too low, this may result in a false negative. Ideally, Doppler interrogation should compare both sides in the same color box if possible.
2. Large hydroceles may limit visualization. Remember to image from different angles and consider repeat imaging after resolution of the hydrocele to ensure that small lesions are not missed.
3. Hematoceles may mimic solid masses. Correlate with a history of trauma and remember to use Doppler imaging to assess for vascularity. Repeat imaging can help assess interval change.
4. Do not dismiss all small avascular lesions. While some of these may be benign, a small lesion can potentially represent a developing malignant neoplasm. If there are suspicious features, remember to correlate with neoplastic serum markers such as alpha fetoprotein and human chorionic gonadotropin. Alternative imaging modalities such as MRI can potentially help troubleshoot difficult cases.

## Supplementary Material

tqag063_Supplementary_Data
