# Supplementary figures and images for: A sound approach to stay on the ball—a review of scrotal pathologies on ultrasound imaging
Source: Br J Radiol. 2026 Mar 17;99(1181):847–58. doi: 10.1093/bjr/tqag063 (PMC13134857; doi:10.1093/bjr/tqag063)

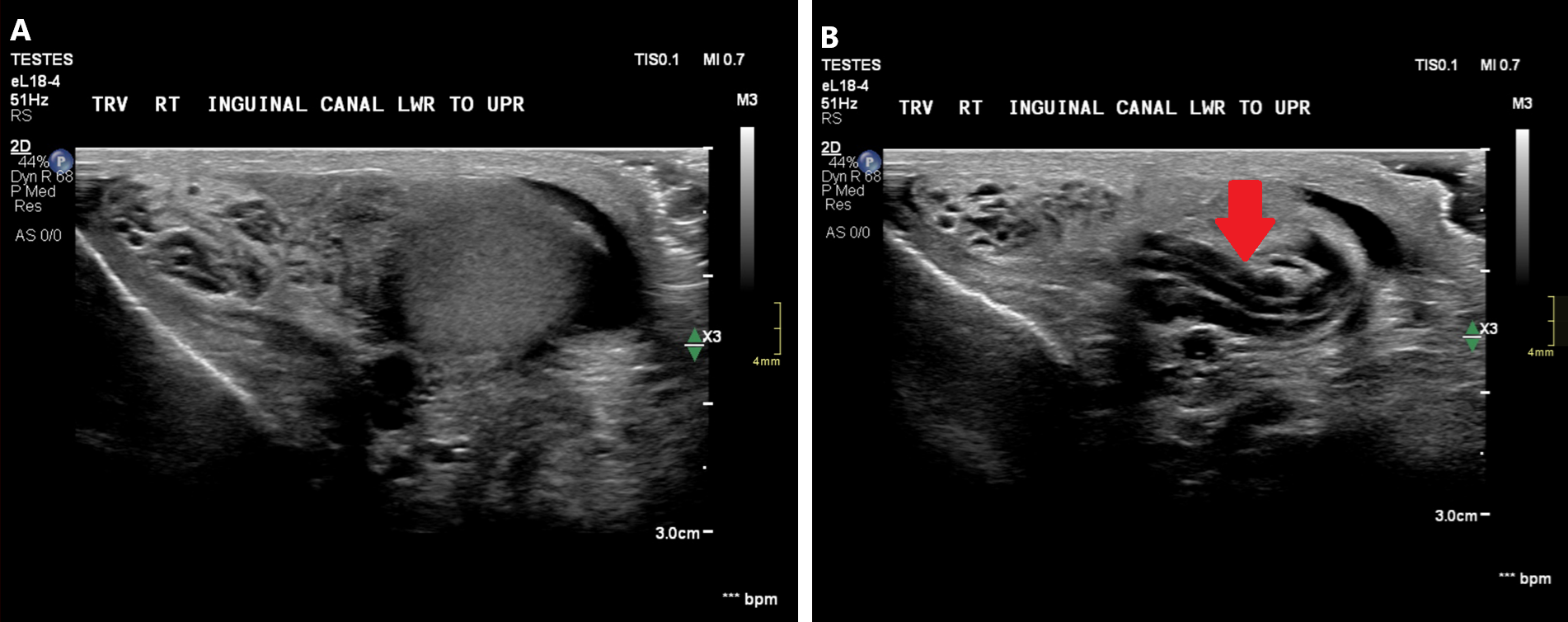

Supplement: tqag063_Supplementary_Data [file tqag063_supplementary_data.zip › Supplementary Figure 3. Bell Clapper.png]

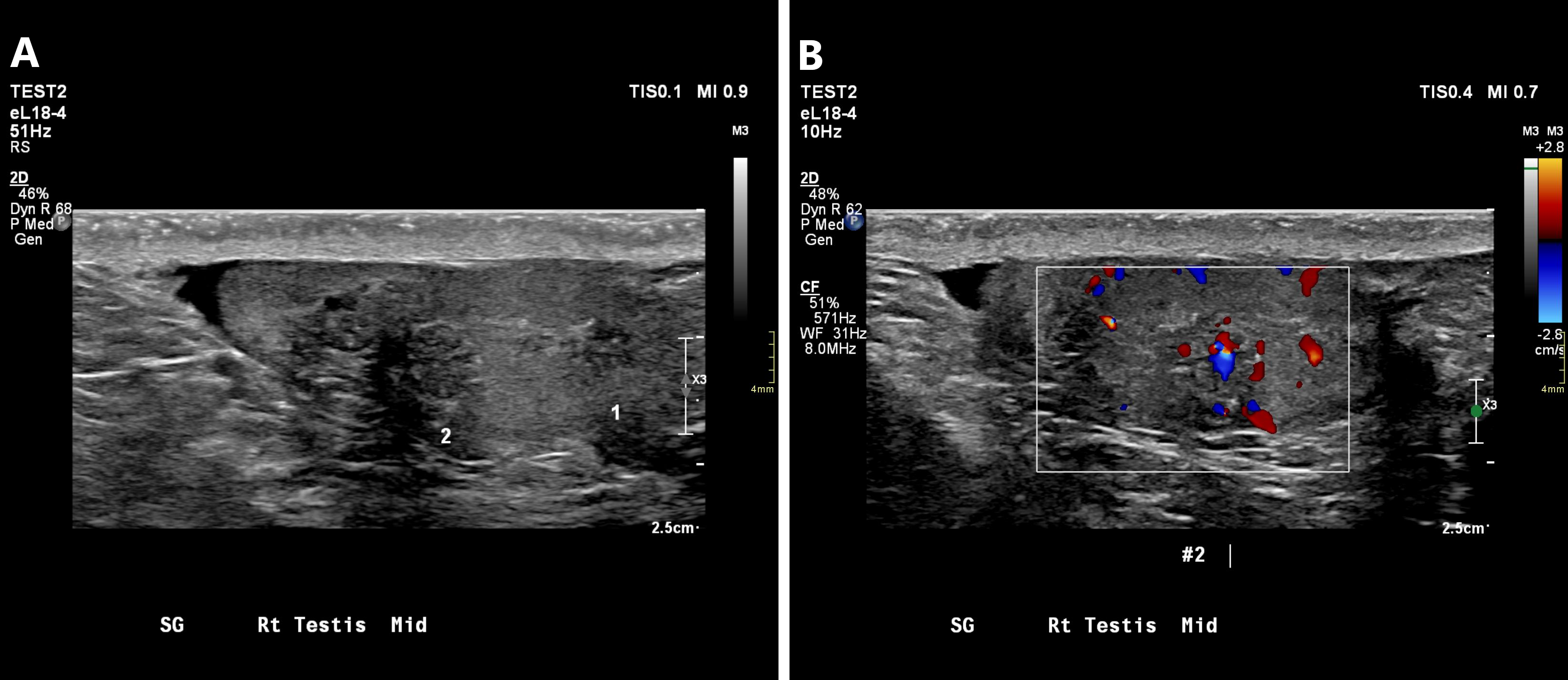

Supplement: tqag063_Supplementary_Data [file tqag063_supplementary_data.zip › Supplementary Figure 4. Adrenal rest..png]

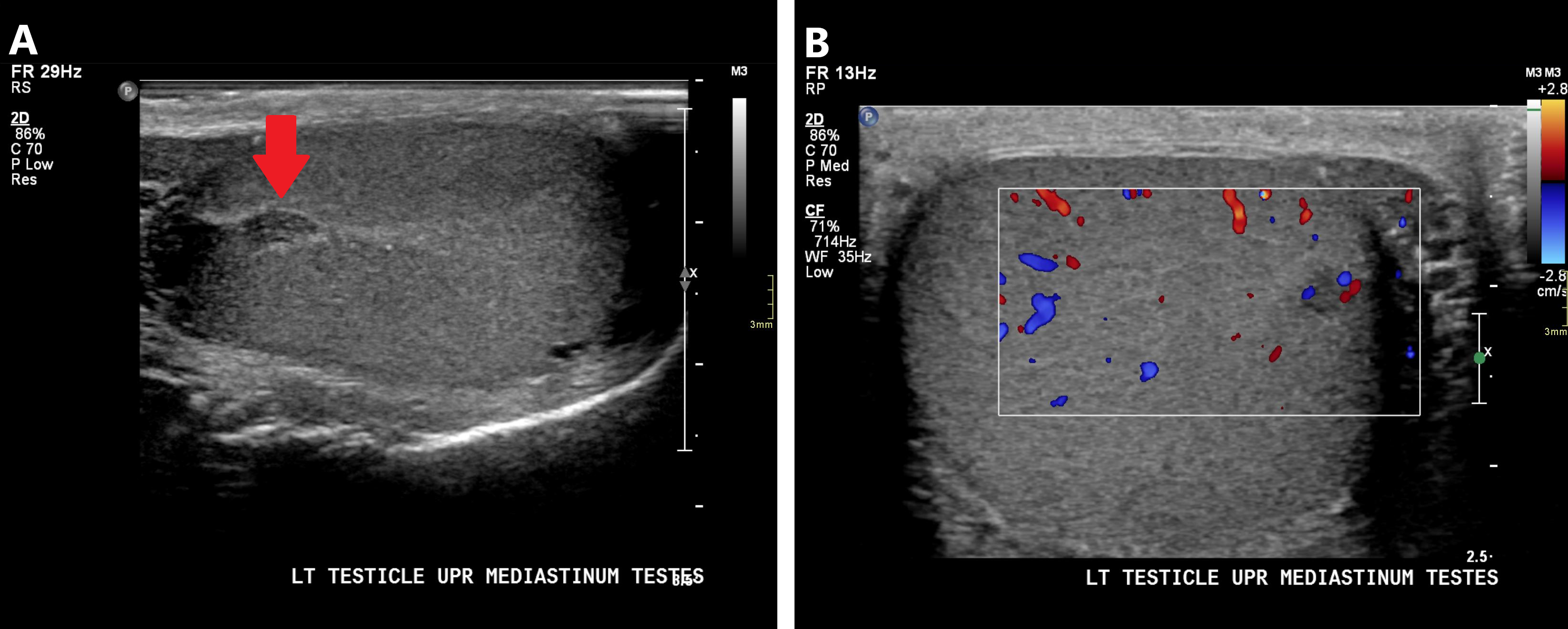

Supplement: tqag063_Supplementary_Data [file tqag063_supplementary_data.zip › Supplementary Figure 1. Rete testis..png]

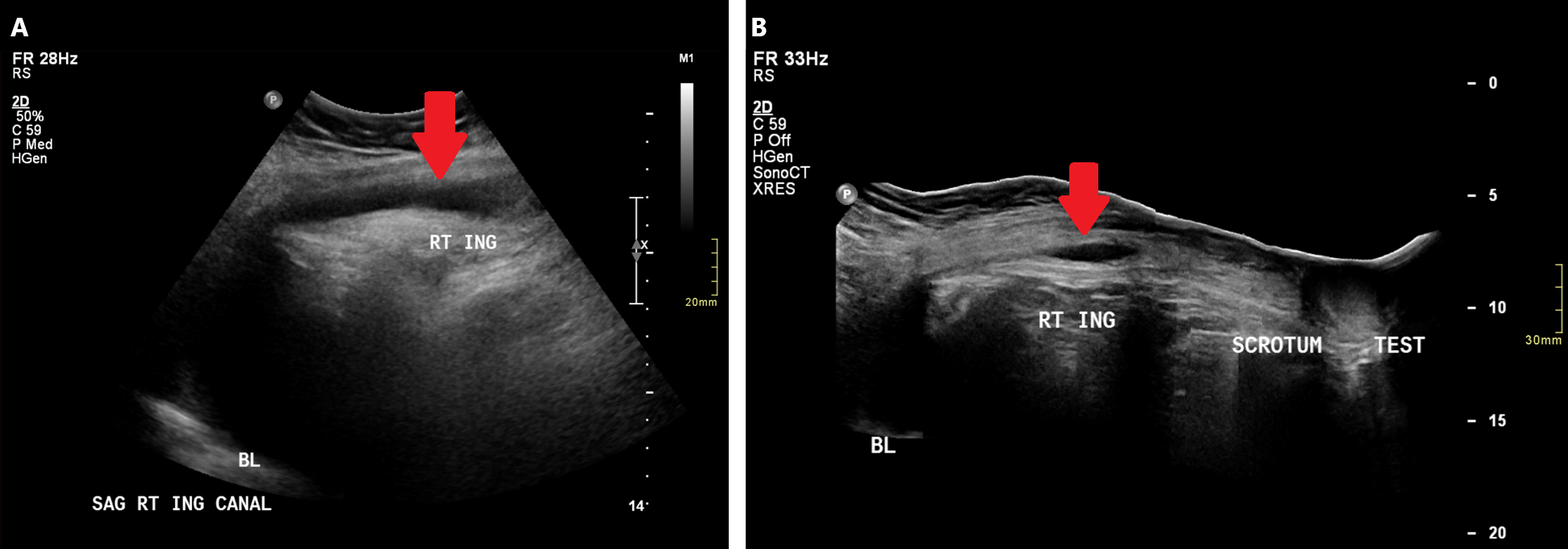

Supplement: tqag063_Supplementary_Data [file tqag063_supplementary_data.zip › Supplementary Figure 2. inguinal hernia..png]
